# The Protein Kinase C Agonist PEP005 (Ingenol 3-Angelate) in the Treatment of Human Cancer: A Balance between Efficacy and Toxicity

**DOI:** 10.3390/toxins2010174

**Published:** 2010-01-22

**Authors:** Elisabeth Ersvaer, Astrid Olsnes Kittang, Peter Hampson, Kristoffer Sand, Bjørn Tore Gjertsen, Janet M. Lord, Øystein Bruserud

**Affiliations:** 1 Section for Hematology, The University of Bergen, Bergen, Norway; Email: elisabeth.ersvar@med.uib.no (E.E.); astrid.Olsnes@med.uib.no (A.O.K.); kesand@gmail.com (K.S.); bjorn.gjertsen@med.uib.no (B.T.G.); 2 Division of Hematology, Department of Medicine, Haukeland University Hospital, Norway; 3 School of Immunity and Infection, Birmingham University Medical School, Birmingham, UK; Email: pxh048@bham.ac.uk (P.H.); J.M.Lord@bham.ac.uk (J.M.L.)

**Keywords:** cancer-protein kinase C-PEP005

## Abstract

The diterpene ester ingenol-3-angelate (referred to as PEP005) is derived from the plant *Euphorbia peplus*. Crude euphorbia extract causes local toxicity and transient inflammation when applied topically and has been used in the treatment of warts, skin keratoses and skin cancer. PEP005 is a broad range activator of the classical (α, β, γ) and novel (δ, ε, η, θ) protein kinase C isoenzymes. Direct pro-apoptotic effects of this drug have been demonstrated in several malignant cells, including melanoma cell lines and primary human acute myelogenous leukemia cells. At micromolar concentrations required to kill melanoma cells this agent causes PKC-independent secondary necrosis. In contrast, the killing of leukemic cells occurs in the nanomolar range, requires activation of protein kinase C δ (PKCδ) and is specifically associated with translocation of PKCδ from the cytoplasm to the nuclear membrane. However, in addition to this pro-apoptotic effect the agent seems to have immunostimulatory effects, including: (i) increased chemokine release by malignant cells; (ii) a general increase in proliferation and cytokine release by activated T cells, including T cells derived from patients with chemotherapy-induced lymphopenia; (iii) local infiltration of neutrophils after topical application with increased antibody-dependent cytotoxicity; and (iv) development of specific anti-cancer immune responses by CD8^+^ T cells in animal models. Published studies mainly describe effects from *in vitro* investigations or after topical application of the agent, and careful evaluation of the toxicity after systemic administration is required before the possible use of this agent in the treatment of malignancies other than skin cancers.

## 1. Introduction

A wide range of carcinogenesis-associated molecules are now investigated as possible therapeutic targets in human malignancies. These possible targets are usually mediators that show an altered expression in cancer cells or they affect essential cancer cell functions, e.g., regulation of proliferation or viability [1,2]. The pharmacological agents investigated can be either molecules known to, or designed to interact with the possible targets. An alternative strategy is to search for new anticancer agents in preparations used in traditional medicine, identify the active compound(s) and characterize their molecular effects [3].

PEP005 (ingenol 3-angelate) is derived from the plant *Euphorbia peplus* and crude euphorbia extracts have been used for centuries in traditional Thai and Australian medicine for treating various skin conditions, including warts, keratoses and cancers [3,4]. Fractionation of the sap yielded several macrocyclic diterpenes with cytotoxic activity or the ability to influence cellular differentiation, and ingenol-3-angelate thus emerged as a possible anti-cancer agent. This hydrophobic diterpene ester is now referred to as PEP005; it is strongly cytotoxic at high concentrations (100 µg/mL) [3] and at lower concentrations of 10-100 ng/ mL it is a selective activator of Protein kinase C (PKC) [4].

## 2. The Protein Kinase C Family

### 2.1. Classification and Characterization of Protein Kinase C Isoenzymes

The PKC family was first distinguished by their status as cyclic nucleotide-independent kinases [5,6] and is now a complex family of at least 11 phospholipid-dependent serine/threonine kinases with distinct functions and tissue distribution [7,8,9,10]. PKC isoenzymes consist of a single polypeptide chain with a C-terminal kinase domain and a regulatory *N*-terminal domain that interacts with phosphatidylserine, Ca^2+^, diacylglycerol, phorbol ester and/or other lipids [9]. The PKCs can be activated by a wide range of signals, including release of second messengers during lipid-mediated signaling, other signaling pathways like the PI3K-pathway, direct molecular binding to for example ceramide, crosstalk between PKC isoenzymes, reactive oxygen species or proteolytic cleavage by caspases (for references see [8]). PKCs have been regarded as possible participants in carcinogenesis, even though PKC mutations are very uncommon in human cancers [8]. Members of the PKC family are classified as conventional, novel or atypical PKCs, depending upon their co-factor requirements (Table 1) [7]. It can also be seen from the table that the various PKCs have different effects on apoptosis, though most isoenzymes have anti-apoptotic effects [11]. 

**Table 1:** Classification of PKC isoforms (8-11).

	Classical isoforms cPKC	Novel isoforms nPKC	Atypical isoforms aPKC
**Members**	α, β_I_, β_II_, γ	δ, ε, η, μ, θ	ζ, ι/λ
**Phorbol ester activation**	Yes	Yes	No
**Regulatory cofactors**	Diacylglycerol	Diacylglycerol	Independent of Ca and diacylglycerol
	Phosphatidyl-serine	Ca-independent	
	Ca		
**Effect on apoptosis**	Antiapoptotic: α, β_I_, β_II_	Antiapoptotic: ε	Antiapoptotic: ζ
		Proapoptotic: δ	

Although PKC mutations are very uncommon in human cancers, the expression of various PKCs, including PKCδ, is often altered in human cancers, as illustrated by the data summarized in Table 2 [12,13,14,15,16,17,18,19,20,21,22,23,24,25,26,27,28,29,30,31,32,33,34,35,36]. Under physiological conditions triggering of phospholipase C activation leads to increased Ca^2+^ and diacylglycerol levels in the cell. These mediators can activate PKC, leading to a wide range of cellular events, depending on the isoenzyme activated [37,38]. In cancer, PKCα and β have been linked to increased invasion, proliferation, drug resistance and genetic instability [37], and like PKCε, they are thought to be oncogenes [39]. PEP005 is a PKC agonist primarily achieving its pro-apoptotic effects through PKCδ, but its effects on intracellular signaling networks will also be influenced by the levels and activation of the other PKCs.

**Table 2:** Altered PKC expression in human cancers.

**PKC isoform**	Tumor Type	Expression	References
*Classical*			
**PKC-α**	Bladder	Increased	[12]
	Brain	Decreased	[13]
	Brain	Increased	[14]
	Breast	Decreased	[15,16]
	Ovarian	Decreased	[17]
	Renal	Decreased	[18]
	Colon	Decreased	[19]
	T-cell leukemia	Decreased	[20]
**PKC-β**	Bladder	Decreased	[12]
	Colon	Decreased	[21,22,23]
	Prostate	Decreased	[24]
	T-cell leukemia	Decreased	[20]
	Melanoma	Decreased	[25]
**PKC-βI**	Bladder	Decreased	[26]
**PKC-βII**	Bladder	Decreased	[27]
	Colon	Decreased	[28]
	DLBCL	Increased	[29]
*Novel*			
**PKC-δ**	Bladder	Decreased	[12,26,27]
	Brain	Decreased	[14]
	Colon	Increased	[23]
	Squamous cell carcinoma	Decreased	[30]
**PKC-ε**	Bladder	Increased	[12]
	Brain	Increased	[31]
	Breast	Increased	[32]
	Colon	Decreased	[23]
	Prostate	Increased	[24]
	Thyroid	Decreased	[33]
**PKC-η**	Breast	Decreased	[34,35]
	Colon	Decreased	[21]
	Renal	Increased	[18]
**PKC-θ**	Gastrointestinal stromal tumor	Increased	[36]

### 2.2. PKCδ and the Effects of PEP005

PEP005 is an activator of novel and classical PKC isoenzymes but its pro-apoptotic effects in leukemic cells rely upon the activation of PKCδ and its translocation from the cytoplasm to the plasma membrane, nuclear membrane and mitochondrial membrane in CHO-K1 cells and AML cell lines [4]. PKCδ activation can slow cell proliferation, induce cell cycle arrest and enhance differentiation in various undifferentiated cell lines. It also promotes apoptosis of malignant cells through: (i) activation of caspases and (ii) increased stability of p53 due to activation of IκB-kinase and thereby increased p53 protein expression despite reduced p53 transcription [37,39,40,41]. In addition to these functions, PKCδ has been reported to phosphorylate up to 10 different signaling molecules, in addition to mitochondrial and nuclear proteins [39,40,41,42,43,44,45,46,47,48,49,50,51]. For example, it has been shown that in response to apoptotic stimuli such as cytarabine, PKCδ translocates to the nucleus where it co-localizes with and phosphorylates lamin B leading to dissolution of the nuclear lamina, and that this could be reduced following PKCδ inhibition. These signals can result in a broad variety of cellular effects, together supporting the hypothesis that PKCδ activity plays a role in regulating the balance between cell proliferation and apoptosis [38,39,40,41,42,43,44,45,46,47,48,49,50,51].

PKCδ^-/-^ mice develop normally and are fertile, suggesting that PKCδ plays minor roles during development, or that its actions can be taken over by another PKC isoenzyme [42,48]. In contrast, PKCδ seems to play important roles in normal hematopoiesis and oncogenesis. PKC isoforms α, βI, δ, ε, ζ and η are all expressed in myeloid cells [38]. Recently, *in vitro* studies have suggested that PKCδ together with PKCα can be essential for monocyte differentiation [42,43]. The human PKCδ gene is located on the short arm of chromosome 3 in a region where there is loss of heterozygosity in many epithelial cancers, suggesting that down regulation of PKCδ contributes to tumor progression [41,43,44,51]. On the other hand, elevated PKCδ expression has been described in multiple myeloma [40], and overexpression of phosphorylated-PKC is found in nearly half of acute myelogenous leukemia (AML) patients [42]. 

**Figure 1:**
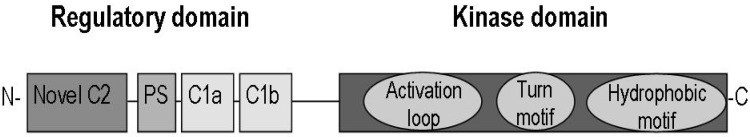
The molecular structure of PKCδ (adapted from [45]). The molecule has a regulatory and a kinase domain. The Novel C2 domain of the regulatory part is Ca^2+^ insensitive in contrast to the conventional PKC’s C2 domains. The C1a and C1b parts can bind diacylglycerol (DAG) as well as phorbol esters. The pseudosubstrate (PS) domain has structural similarities to the substrate of the kinase domain and binds to the active site of the kinase domain. Binding of C2 and C1 to membrane structures will release the PS domain from the active site and make substrate binding possible. The Hinge domain is the cleavage site for Caspase 3, this cleavage occurs in the nucleus and results in the release of the δ-catalytic fragment (δCF) that corresponds to the kinase domain. Phosphorylation of several tyrosine and serine residues both in regulatory and kinase domain has been described. The overall phosphorylation pattern determines the intracellular compartmentalization of the enzyme. Among the kinases involved in phosphorylation of PKCδ are the non-receptor tyrosine kinases Abl and Src like kinase-Lyn.

**Figure 2:**
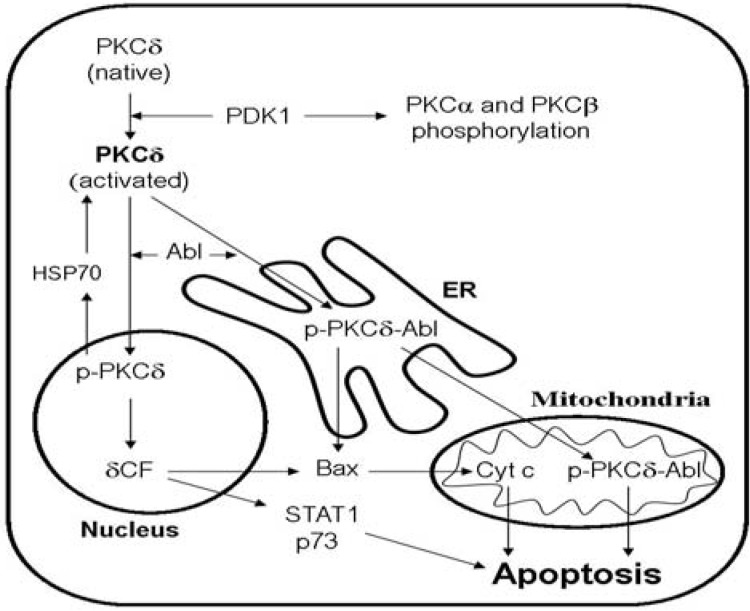
Intracellular compartmentalization of PKCδ. Phosphoinositide dependent kinase 1 (PDK1) is responsible for the initial activating phosphorylation of PKCδ; this enzyme can also phosphorylate PKCα and PKCβ as an initial activating event for these enzymes. If a pro-apoptotic signal is involved the activated PKCδ is thereafter translocated either to the nucleus or to the endoplasmic reticulum (ER). After nuclear translocation caspase 3 cleavage results in the formation of the δCF fragment that has a pro-apoptotic effect either (i) *via* upregulation of Bax and subsequent mitochondrial release of cytochrome c, or (ii) *via* the cytoplasmatic mediators STAT1 and p73. Alternatively, the activated PKCδ can be exported from the nucleus by a mechanism involving dephosphorylation and subsequent molecular stabilization by Heat shock protein 70 (HSP70). The translocation to ER is initiated through cytoplasmatic association of activated PKCδ with the Abl kinase; this results in PKCδ phosphorylation and translocation of the p-PKCδ-Abl complex to ER where pro-apoptotic signaling is initiated either through Bax or through further translocation of the complex to the mitochondria [11,45,46,47].

The molecular structure of the PKCδ isoenzyme is shown in Figure 1 [45]. The intracellular compartmentalization of PKCδ depends upon its post-transcriptional modulation, and PKCδ-mediated signaling has pro-apoptotic effects through several pathways (Figure 2) [11,46,47]. Briefly, translocation of the enzyme from the cytoplasm to the nucleus seems crucial to its pro-apoptotic actions. Initial phosphorylation of the enzyme on tyrosine residues occurs in response to apoptotic stimuli and activated PKCδ accumulates in the nucleus together with activated caspase 3. PKCδ is cleaved by this caspase and a catalytic fragment is thereby formed. This fragment has constitutive activity, remains in the nucleus and induces apoptosis possibly through phosphorylation of apoptosis-regulating proteins. An alternative pro-apoptotic pathway is mediated through the endoplasmic reticulum and mitochondria (Figure 2).

Several PKC isoenzymes show altered expression in human cancers as summarized in Table 2 [12,13,14,15,16,17,18,19,20,21,22,23,24,25,26,27,28,29,30,31,32,33,34,35,36]. Moreover, as some of these isoenzymes can have anti-apoptotic effects, whereas PKCδ is regarded as an important pro-apoptotic mediator, the ultimate outcome of altered expression will depend upon the balance between the activity of pro- and anti-apoptotic PKCs. [11,46,47]. In these models PKCα and PKCβ had anti-apoptotic effects, and suppression of these enzymes caused induction of apoptosis with upregulation of pro-apoptotic PKCδ [46]. However, overexpression of PKCδ alone was not sufficient for induction of apoptosis. These observations clearly illustrate that the crosstalk between pro- and anti-apoptotic PKC isoforms is important, and the final effect of the PKC-agonist PEP005 may therefore depend upon the balance between the various isoenzymes present within a tumor and the drug may be less effective in those tumors with increased levels of the anti-apoptotic isoforms (Table 2).

### 2.3. The Phenotype of PKCδ Null Mice

As stated above PKCδ^-/-^ mice develop normally and are fertile [42,43,44]. However, studies using PKCδ null mice (PKCδ^-/-^) have given important insight into the role of PKCδ *in vivo*. Studies by Leitges and colleagues showed that vein segments from PKCδ^-/-^ mice, subsequently grafted onto the carotid arteries of recipient mice (either PKCδ^-/-^ or PKCδ^+/+^), lead to more severe atherosclerosis than was seen with PKCδ^+/+^ vein grafts [44]. The authors went on to show that atherosclerotic lesions in PKCδ^-/-^ mice contained significantly more smooth muscle cells (SMCs) than were found in the wild-type animals, and that this increased number of cells correlated with decreased SMC death in the lesions of PKCδ^-/-^ mice [44]. Finally, the authors demonstrated that SMCs from PKCδ^-/-^ mice were resistant to cell death after treatment with a number of apoptosis-inducing stimuli, including UV light, H_2_O_2_, and TNF-α [44]. A more recent study by Humphries and colleagues showed that γ-irradiation induced apoptosis of parotid glands was reduced by 60% in PKCδ^-/-^ mice when compared to wild-type mice [48]. It was shown that primary parotid cells from PKCδ^-/-^ mice were defective in mitochondrial dependent apoptosis, as shown by a suppression of etoposide-induced cytochrome-c release. Moreover, apoptotic responsiveness was restored by re-introduction of PKCδ [49]. Both of these studies demonstrate a pro-apoptotic role of PKCδ *in vivo*. Other work with PKCδ^-/-^ mice has demonstrated a role for PKCδ in the negative regulation of B-cell proliferation [50]. In this study, mice that lacked PKCδ exhibited an expansion of B-lymphocytes leading to the formation of germinal centres in the absence of stimulation, and the rate of proliferation of B-lymphocytes in response to stimulation was greater in the PKCδ^-/-^ mice [49,50]. Similar studies showed that PKCδ deficiency prevented B-cell tolerance, allowing maturation and terminal differentiation of self reactive B-cells in the presence of tolerizing antigens [50]. Whether this was due to diminished apoptosis was not investigated. 

## 3. The Importance of Neutrophil Recruitment and Humoral Immunity after Topical Application of Pep005 for Skin Cancer

Human neutrophils express the conventional PKCs α, βI, βII, the novel PKC δ and the atypical PKCζ, and PKCs are important in neutrophil function [9]. PKC is involved in the activation of integrins as well as other adhesion molecules; it associates with several cytoskeletal components and thereby forms a functional bridge between the plasma membrane and the cytoskeleton. 

### 3.1. PEP005 EFFECTS on Endothelial Cells

The recruitment of neutrophils to sites of inflammation usually occurs across the endothelial cells in postcapillary venules [52,53,54]. PEP005 induces the expression of IL1β, TNF-α and the neutrophil chemotactic chemokine CXCL8 in mouse normal skin and skin tumors as well as in human keratinocytes, fibroblasts and melanoma cells [55]. These cytokines may then: (i) activate neighboring endothelial cells and thereby favor adhesion and transendothelial migration of circulating leukocytes; and (ii) create a chemotactic CXCL8 gradient that favors local recruitment of neutrophils [56]. 

An additional mechanism for PEP005-induced recruitment of neutrophils could be direct effects on the endothelial cells with increased expression of adhesion molecules and/or the induction of neutrophil-chemotactic cytokines. A recent study described increased transcriptional upregulation of the expression of E-selectin, ICAM-1 and CXCL8 in umbilical vein endothelial cells after exposure to PEP005 [56]. When using a flow-based adhesion assay PEP005 then caused increased adhesion of neutrophils to a level that was comparable to endothelial cells activated with TNF-α. The adhesion was dependent on E-selectin, was accompanied by a translocation of PKCδ from the cytosol to the perinuclear membrane, and siRNA knockdown of PKCδ abolished neutrophil recruitment [56]. 

Taken together these results suggest that PEP005 causes local recruitment of neutrophils through: (i) direct effects on endothelial cells with increased adhesion; (ii) indirect effects on the endothelial cells through local release of activating cytokines from neighboring cells; and (iii) the release of neutrophil chemotactic CXCL8 by endothelial cells and perivascular cells. Although one cannot exclude that umbilical cord and microvascular endothelial cells show functional differences, it seems likely that all three mechanisms contribute to the inflammatory response to topical application of PEP005.

### 3.2. The Anticancer Effect of Neutrophils after Topical Application of PEP005

Topical treatment of skin tumors with PEP005 induces cancer cell necrosis followed by local inflammation characterized by neutrophil infiltration and release of reactive oxygen species [55]. The treatment also increases the levels of antitumor antibodies and thereby enhances tumor cell killing by antibody-dependent neutrophil cytotoxicity. The following observations have been made after implantation of tumor cells in mice followed by topical PEP005 treatment [3,55]:

- Topical application of PEP005 can cure implanted skin cancers without later relapse in the T cell deficient Foxn1^nu^ mice. This effect is associated with local macroscopic inflammation due to leukocyte infiltration dominated by neutrophils. After antibody-depletion of neutrophils topical PEP005 treatment caused a similar initial ablation, but tumors later re-emerged.
- The neutrophil extravasation into the inflamed sites is severely impaired in CD18-deficient mice; topical treatment of implanted tumors in these animals was associated with initial cure followed by a weak local inflammation and later tumor relapse.
- NK cells and macrophages are present in Foxn1^nu^ mice, and macrophages are seen in PEP005 induced infiltrates. The local inflammation and relapse rate were not altered by depletion of NK cells. Neither inflammation nor relapse risk was altered for tumors implanted in Csfmop/Csfmop mice that lack functional M-CSF and therefore are severely monocytopenic.
- The effect of topical PEP005 was investigated for LK2 tumors implanted in SCID mice that lack a humoral immune system [55]. Tumors grew at similar rates and the initial tumor-ablative effect and local inflammatory reactions were similar to Foxn1^nu^ mice, but a high relapse rate was observed for the B cell-depleted mice.

**Figure 3:**
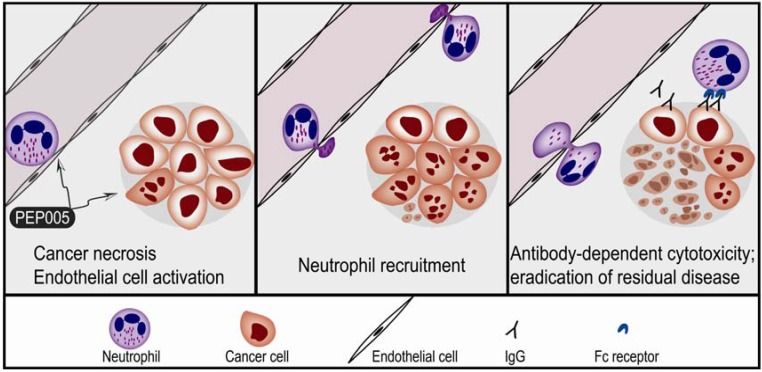
Effects of PEP005 in experimental skin cancer. (LEFT) Topical application of PEP005 causes high local drug concentrations with two direct effects; endothelial cell activation with neutrophil adhesion; and (ii) direct induction of necrosis in the malignant cells. (MIDDLE) There is transmigration and local neutrophil recruitment with a local inflammation. (RIGHT) Finally there is an antitumor humoral immune response leading to antibody-dependent cytotoxicity and eradication of residual cancer cells.

Taken together these results suggest that topical application of PEP005 to skin tumors mediates anticancer effects through three distinct phases (Figure 3). Firstly, the initial tumor ablation is caused by a direct effect of the drug and local production of inflammatory cytokines [55]. The second phase is characterized by local inflammation due to neutrophil infiltration. During the third and last phase tumor-reactive antibodies are induced and relapses are avoided through antibody-dependent neutrophil cytotoxicity that eliminates remaining cancer cells [55].

### 3.3. Clinical Studies of PEP005 in the Treatment of Skin Cancer

Two randomized studies have investigated the short-course use of topical PEP005 in the treatment of actinic keratosis, a premalignant lesion that can progress to invasive squamous cell carcinoma [57,58]. Both studies concluded that topical application was effective and caused by local induction of necrosis and inflammation. The safety profile seems favorable, and treatment-related scarring was not a major problem. Thus, these studies support the conclusions from animal studies that topical PEP005 in the treatment of skin cancer is safe and effective and without systemic toxicity [3].

## 4. Antileukemic Effects of Pep005

### 4.1. Effects of PEP005 on Acute Myelogenous Leukemia Cells

Acute myelogenous leukemia (AML) is the human malignancy where the effects of low-dose PEP005 have been most extensively studied [59,60]. In most of these experiments PEP005 was used at a final concentration of 20 nM. The following effects were described for primary AML cells [59,60]:

- *Chemokine release.* Primary human AML cells show constitutive release of a wide range of chemokines [61,62]. PEP005 causes increased release of both CCL and CXCL chemokines, including CXCL8 that also was released at increased levels by skin cells after topical application (see above). The chemokines released at increased levels are pro-angiogenic and chemotactic not only for neutrophils but also for T cells and monocytes.
- *Chemokine receptor expression.* PEP005 has only minor effects on the expression of most CCR and CXCR receptors (CCR1-3, CCR5, CXCR2, 3), the only exception being CXCR4 that shows decreased expression. CXCR4 is one of the two receptors for the CXCL12 chemokine that is usually not released or only released at low levels by primary human AML cells [61]. However, it is released by bone marrow stromal cells [62]. The CXCL12/CXCR4 system is important for AML cell migration and CXCR4 expression seems to have an adverse prognostic impact in AML [63]. For this reason the PEP005 induced reduction in CXCR4 expression should possibly be regarded as an anti-leukemic effect.
- *Cytokine release.* Other cytokines are also released at higher levels, including Hepatocyte growth factor (HGF) and Granulocyte-macrophage colony-stimulating factor (GM-CSF) [59,60].
- *Differentiation.* PEP005 decreases the expression of stem cell markers (including CXCR4) and increases the expression of lineage-associated markers, an observation consistent with differentiation induction.
- *Apoptosis regulation.* PEP005 increases the expression of Bax and the activation of caspase 3. These pro-apoptotic effects are seen over a wide concentration range, whereas no induction of apoptosis was evident for normal CD34^+^ hematopoietic cells when testing concentrations up to 200 nM.
- *Intracellular signaling.* The effect in AML cells is mediated through a PKCδ agonistic effect. The ERK1/2 pathway then seems to be important for the increased chemokine release together with increased expression of the NFκB subunits p50, p52 and p65.

Thus, the effect of PEP005 at these relatively low concentrations is mediated through induction of apoptosis and differentiation. 

### 4.2. The Role of PKC in Other Leukemias

The effect of PEP005 has been investigated only in AML, but various PKC isoenzymes also seem to be important in other leukemias as reviewed by Redig and Platanias [8]. In chronic lymphocytic leukemia (CLL) several PKCs are expressed in cells from most patients, including PKCβ, PKCγ, PKCδ and PKCζ and for some patients also PKCα, PKCι and PKCε. Global PKC inhibition induces apoptosis in CLL cells. So far there is no evidence for an important role of PKCδ in CLL and PKCα seems to be more important in regulation of proliferation and apoptosis in these cells [64]. Thus, the balance between pro- and anti-apoptotic isoforms may be important not only in solid tumors (Table 2) but also in hematological malignancies. The possible importance of PKC for disease development or chemosensitivity in other leukemias remains to be clarified.

## 5. Effects of PEP005 in Solid Tumors

### 5.1. Pharmacological *in Vitro* Studies

The studies described above demonstrate that PEP005 has an anticancer effect in different malignancies, but it should be emphasized that human cancer cells can also be generally resistant or the pro-apoptotic effect may be context-dependent [65]. In a recent study the effect of PEP005 on TRAIL (Tumor necrosis factor related apoptosis inducing ligand)-induced apoptosis was examined in human melanoma cell lines [66]. Enhancing or inhibitory effects on TRAIL-induced apoptosis were then observed depending on the cell line investigated, and the authors suggested that the effect of PEP005 in these models is not dependent on PKCδ alone but also on low expression of PKCε. 

Another study described induction of senescence in melanoma cells after *in vitro* exposure to PEP005; this additional pharmacological effect was observed for 20% of the cell lines [67]. This growth arrest involved signaling through ERK, the same pathway that seems responsible for the pro-apoptotic and chemokine-increasing effects in AML cells (see above). The growth arrest seen with PEP005 treatment consisted of accumulation of cells in G_1_ phase for up to 24 hours after *in vitro* exposure. Optimal combination of PEP005 with conventional cytotoxic drugs therefore seems to require a lag-time between exposure to the different drugs [67].

Resistance mechanisms to PEP005 have also been investigated in colon cancer cell lines that were cross-resistant to several chemotherapeutics [68]. PEP005 resistance seemed dependent on high expression of the small vasoactive peptide E1 that stimulates proliferation of colorectal cancer cells *via* the ETRA receptor. Other studies in colon cancer cells suggest that PEP005 can affect signaling through several pathways with increased phosphorylation of PKCδ, Raf1, ERK1/2, c-jun, p38, mitogen-activated protein kinase (MAPK) and PTEN [69]. These authors also described that PEP005 reduced the expression of PKCα and reduced the levels of the active phosphorylated form of Protein kinase B. 

Taken together these observations suggest that PEP005 can affect several intracellular signaling pathways and that resistance may occur dependent upon the differential activity of pro- and anti-apoptotic pathways in individual patients and between different malignancies. 

### 5.2. Studies in Animal Models

The effect of topical PEP005 has also been tested for other malignancies after skin implantation in Foxn1^nu^ mice [70]. These experiments demonstrated that PEP005 was effective not only against squamous cell carcinoma cells but also cells derived from human and murine melanoma, murine lung carcinoma, human prostate cancer and human cervical carcinoma [3]. Additional *in vitro* studies demonstrated that the drug could kill human breast cancer cells and T-leukemia cells, and for all these cell types the LD90s seemed comparable. The mechanisms behind the effects seem to be destabilization of endocytosed vesicles followed by endosome disruption with release of calcium into the cytoplasm and thereby mitochondrial swelling, disturbed energy metabolism, loss of mitochondrial membrane potential, rapid plasma membrane perturbation and cell death due to necrosis [3].

### 5.3. The Possibility of Topical Application for Other Cancers

Another possibility for topical treatment is bladder cancer [3]. The experience so far is limited, but *in vitro* studies suggest that normal urothelial cells may be less sensitive than bladder cancer cells. However, initial animal studies will be required because frequent inspection of the local inflammation is not possible in bladder cancer, and if severe hematuria occurred this complication may require specific therapeutic intervention. 

## 6. Immunomodulatory Effects of PEP005

The increased release of chemotactic chemokines that enhance recruitment of various leukocytes by PEP005 treated AML cells must be regarded as an immunostimulatory effect (Figure 3). The effects on neutrophils are described above. In addition PEP005 has direct T cell effects in AML derived cells resulting in: (i) increased proliferative T cell responses in cells from patients with untreated disease and patients with severe chemotherapy-induced panleukopenia, including severe T lymphopenia [3,71]; and (ii) increased release of several cytokines by activated T cells, including IFNγ, GM-CSF, IL-2, IL-10, IL-13 and TNF-α, in cells from AML patients with chemotherapy-induced cytopenia (72). Thus, PEP005 *in vitro* seems to have both anti-leukemic and immunostimulatory effects in cells from AML patients and if this is extended to the *in vivo* situation, the immunostimulation could also include indirect effects through increased T cell recruitment [59] and direct T cell stimulatory effects [71,72].

**Figure 4:**
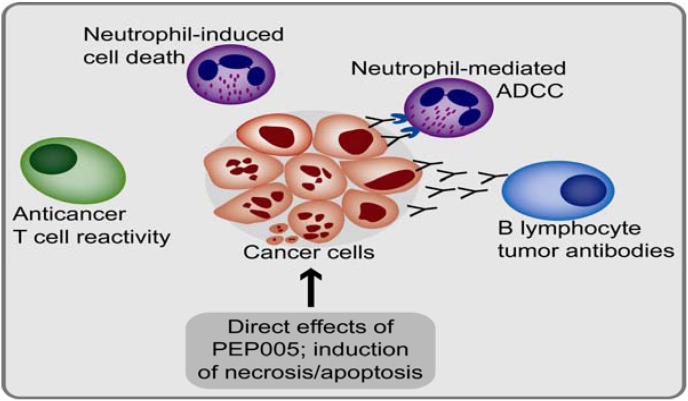
A summary of direct and indirect anticancer effects of PEP005. PEP005 has direct effects on malignant cells leading to either necrosis or apoptosis depending on the drug concentration. High concentrations are relevant for topical application, whereas lower concentrations are more relevant for leukemic disease. The indirect effects that may contribute to the anticancer effects are: (i) increased T cell reactivity, including increased cytokine release; (ii) local recruitment of neutrophils, endothelial cell activation contributes to this; (iii) induction of anticancer humoral immune responses with enhanced antibody-dependent cellular cytotoxicity (ADCC).

Even though the early animal studies concluded that elimination of implanted tumors in mice were dependent on neutrophils and B cells (see above), a recent report reported that specific T cell responses can also be induced following local treatment [73]. Induction of a tumor-specific CD8^+^ response by PEP005 was observed, and this response contributed to regression of distant metastases. PEP005 was also found to have adjuvant properties and upregulated the expression of T cell costimulatory molecules CD80 and CD86 on dendritic cells. These observations further demonstrate that PEP005 has a broad immunostimulatory effect (Figure 4).

## 7. Concluding Remarks: Efficiency *versus* Toxicity in the future Use of PEP005 in Cancer Treatment

The overall literature described above suggests that PEP005 can mediate anticancer effects in different malignancies (Figure 4), but it should be emphasized that except for local application in skin tumors most of the present evidence comes from experimental *in vitro* studies. In contrast, the immunostimulatory effect is documented both in experimental models, *in vitro* studies of human T cells and after topical application in humans. In systemic therapy the immunostimulatory effects represent a beneficial effect with regard to anticancer activity but also a potential risk of toxicity if pro-inflammatory effects predominate. Testing of PEP005 in animal models of leukemia is now required to determine if the compound applied systemically can achieve its anti-leukemic effects without significant toxicity. 

### 7.1. Combination of PEP005 with Conventional Chemotherapy

PEP005 has been used as a single agent therapy in the topical treatment of skin diseases. Preclinical studies suggest that a combination of PKCδ agonists with conventional chemotherapy should be considered in human cancer therapy. PKCδ activation is induced after exposure of leukemia cells to etoposide, this is also observed after exposure of myeloid leukemia cells to Interferon-α, and in addition PKCδ seems important for anthracyclin-induced pro-apoptotic signaling [74,75]. Both etoposide and anthracyclines are widely used in the treatment of several other malignancies, including lymphomas and solid tumors, and combination therapy may therefore result in additive or supra-additive effects.

### 7.2. The proinflammatory Effects of PEP005, A Possible Risk during Systemic Therapy

The proinflammatory effect of PEP005 is clearly seen after topical therapy and involves neutrophils, B cells and T cells. Generally, great care should be taken if a drug with known proinflammatory effects is tried in systemic therapy. A dramatic example was the monoclonal antibody TGN1412, an anti-CD28 specific IgG_4_ antibody [76,77,78,79]. Its preclinical screening showed no evidence for severe proinflammatory reactivity, but the phase I study in healthy volunteers resulted in severe multiorgan failure within hours after administration. The pathogenesis was massive cytokine release. Similar reactions have also been observed in other patients and with other agents, e.g., the use of the CD20 specific antibody rituximab in the induction treatment of patients with lymphocytic leukemia [80]. Thus, such reactions are not specific for the TGN1412 antibody but can also be seen with other agents. PEP005 is a drug with known proinflammatory effects, and for these reasons systemic administration of PEP005 has to be done with great care.

The clinical studies of topical PEP005 therapy showed no evidence for systemic effects. However, when using topical application to other body surfaces the risk of increased absorption must be considered. As an example, PEP005 is now considered for the treatment of bladder cancer [3]. The absorption from a relatively large urothelial surface may differ from the skin, and the risk of systemic effects has to be considered in the design of future clinical studies. 

The increased chemokine release by AML cells after PEP005 exposure will also affect NK cells that express receptors for several CCL and CXCL chemokines [81]. It is not known whether this potentially proinflammatory effect will increase the risk of systemic toxicity when tried in AML. 

### 7.3. Cancer-Directed Delivery of PEP005 in Systemic Therapy

One possible approach to avoid severe adverse events during systemic administration of proinflammatory drugs could be to direct the drug release towards the disease compartment. Several strategies may then be possible. In hematological malignancies the disease is usually detected throughout a large part of the body. These disorders often infiltrate diffusely throughout the bone marrow or affect several lymph node regions. Coupling of drugs to a disease-reactive monoclonal antibody has been used to direct anticancer therapy, e.g., coupling to antibodies against the myeloid marker CD33 or the CD20 lymphoid marker [82,83,84]. Another possibility is to administer the drug in a form where drug release is only seen after therapeutic intervention; examples are drug release in a visualized tumor though local ultrasound treatment or photochemical therapy [85,86].

### 7.4. Sequential Treatment with Intensive Chemotherapy and PEP005; Decreased Risk for Proinflammatory-Induced Adverse Events?

Another possibility to avoid adverse events due to proinflammatory effects would be to administer the drug to severely immunocompromised cancer patients. Patients receiving treatment for acute leukemia develop a period of 2-3 weeks with severe leukopenia, and the risk of developing severe side effects may be less in such patients [87]. Furthermore, conventional cytotoxic drugs are often most effective against proliferating cells, and due to its antiproliferative effects PEP005 should possibly be administered sequentially with conventional chemotherapy to achieve a maximal anticancer effect. However, it should be emphasized that even patients with severe chemotherapy-induced cytopenia have an operative immune system [88], and a risk of proinflammatory side effects, though much reduced, is probably present even in such patients.

### 7.5. PEP005 effects on the Chemokine System-Advantage or Disadvantage?

Several chemokines that show altered release after PEP005 exposure have angioregulatory effects [59], but whether the drug will have pro- or anti-angiogenic effects will probably depend on several factors including: (i) additional local chemokine release by stromal cells, (ii) the overall cancer cell chemokine release profile, (iii) genetic polymorphisms within chemokine or chemokine receptor genes; and (iv) the concomitant expression of chemokine decoy receptors [59,61,89,90,91,92,93]. Matrix metalloproteinase (MMP) 2, 9 and 10 can also be constitutively released by primary human AML cells and may also contribute to leukemia-associated bone marrow angiogenesis, but PEP005 has only minor effects on this release [94].

The increased chemokine release may have proinflammatory effects that may represent a risk of toxicity. However, in certain clinical contexts the combination of anticancer and proinflammatory effects may be an advantage. Antileukemic immune reactivity is important for the reduced relapse risk after allogeneic stem cell transplantation, and increased T cell reactivity may then strengthen this antileukemic effect [90,93,95]. Whether modulation of the chemokine system will alter humoral immune reactivity is not known [90,96]. Finally, the possible leukemia-enhancing effect by increased CXCL12/CXCR4 expression may be counteracted by specific inhibitors [97], and this may become true also for other chemokines/chemokine receptors.

### 7.6. Final Comment

PEP005 has both anticancer and proinflammatory effects (Figure 4). These dual effects are an advantage in topical skin application, but it is not known whether the proinflammatory effects will represent an advantage or a disadvantage with risk of severe systemic toxicity after systemic therapy. Only extensive preclinical evaluation in relevant experimental models and careful design of clinical studies can clarify whether systemic use of this drug will be acceptable with regard to the risk of toxicity. 
